# Knowledge, Attitude, and Practice of Dry Eye Treatment by Institutional Chinese Physicians in Singapore

**DOI:** 10.1100/2012/923059

**Published:** 2012-11-08

**Authors:** Wanwen Lan, Sze Yee Lee, Man Xin Lee, Louis Tong

**Affiliations:** ^1^Singapore Eye Research Institute, 11 Third Hospital Avenue, Singapore 168751; ^2^Yong Loo Lin School of Medicine, National University of Singapore, Singapore 117597; ^3^Duke-NUS Graduate Medical School, Singapore 169857; ^4^Singapore National Eye Centre, Singapore 168751

## Abstract

Dry eye is a common health problem worldwide, causing significant discomfort and inconvenience to sufferers. The conventional treatment of dry eye via topical administration of eye drops is deemed palliative and unsatisfactory to many. Traditional Chinese medicine (TCM) has shown some promise in dry eye treatment; however, the extent of its use and acceptance is uncertain. We evaluated the knowledge, attitude, and practice of institutional TCM practitioners in the treatment of dry eye in Singapore. A questionnaire was generated to address the study aims and sent to TCM practitioners listed in the Singapore TCM practitioners' board database. About three quarters of respondents thought that dry eye was not severe enough to be a public health burden but most thought that TCM was effective in the treatment of dry eye. Acupuncture and herbal medicine were most commonly used TCM modalities in dry eye treatment, and a single TCM treatment session would be charged S$20–50 by the practitioner. The majority of surveyed institutional TCM practitioners in Singapore believe that TCM is relevant in dry eye treatment. Public awareness should be raised regarding the availability of TCM as alternative medicine for dry eye.

## 1. Introduction

Dry eye is a highly prevalent disorder, affecting 14% to 33% of the adult population worldwide, does not have an effective therapy, and causes significant loss of productivity at work [1–3]. It incurs significant socioeconomic burden in many societies and is more common in Asia [4]. Dry eye aetiology includes diminished tear production and increased evaporation of tears [5]. This may in turn result in decreased ability to perform daily activities thus having an impact on quality of life [6]. 

Current clinical treatments available for evaporative dry eye include tear supplements, for example, lubricants, tear stimulation and anti-inflammatory medication, oral supplements and tear retention methods: for example, punctal occlusion using cautery or punctum plugs, moisture chamber spectacles/shields, contact lenses, and tarsorrhaphy [7]. Essentially, dry eye can be a lifestyle problem [3]; prolonged gazing and reduced blinking due to activities such as reading and exposure to air-conditioning can result in instability of the tear film [4]. There is a need for more than one modality of treatment for mild-to-moderate dry eye patients, apart from the usage of topical eye drops. Indeed only a small portion of dry eye sufferers use artificial tears regularly [8]. These findings taken together suggest that a combinatorial holistic approach to the management of dry eye may be preferred. 

Traditional Chinese medicine (TCM) is a system of health care originating from China. It views the human body as a microcosm of the world and aims to restore harmony and wholeness within the entity. TCM encompasses various treatment modalities, including acupuncture, herbal medication, tuina (massage), cupping, and moxibustion. With the migration of ethnic Chinese into Singapore in the 19th century, TCM practice has been integrated into local culture and tradition. In 2000, the TCM Practitioners Act was enforced to regulate the practice of TCM in Singapore, as well as to uphold professional conduct and ethics. All TCM practitioners must be registered with the board and pass a qualifying board exam in order to be licensed to practice. The control of Chinese Medicinal Materials is also overseen by the Health Sciences Authority of Singapore, the agency for regulating drugs in Singapore.

The recent years saw an increase in interest in alternative and complementary medicines worldwide [9, 10]. Despite the fact that national health insurance endorsed by the government only encompasses conventional medicine [11], a population-based survey conducted in Singapore showed that 76% (95% C.I. 73.9–77.9%) of participants have used alternate medicine over a one-year period [12]. As high as 88% of these participants used TCM as alternative medicine, and the use of alternative medicine in Singapore was independent of age, income, or education level [12]. In a study involving Singapore children, as many as 80.3% of parents used TCM and western medication concurrently for their children [13]. The increasing popularity of TCM in Singapore (http://www.chinadaily.com.cn/bizchina/2010-08/26/content_11208103.htm) was also shown by the inclusion of conventional physicians in Singapore as registered acupuncturists in the TCM board. Furthermore, TCM departments are being set up even in conventional hospital campuses since 2004, offering a combination of acupuncture and herbal therapies [14]. 

Recently, randomised controlled studies in the use of TCM in dry eye have been published, with some favourable results [15–21]. In one review of 6 randomised controlled trials, a meta-analysis showed that acupuncture significantly improved tear break-up times (*P* < 0.0001), Schirmer's test (*P* < 0.00001) and cornea fluorescein staining (*P* = 0.0001) [22]. Even more recently, a study showed that in the sham acupuncture group (*n* = 21), the tear break-up time changed from 3.71 ± 1.38 s to 4.00 ± 1.34 s (change not statistically significant) after 3 weeks, whereas in the acupuncture treatment group (*n* = 21), it significantly increased from 3.29 ± 1.01 s to 4.24 ± 1.26 [23, 24]. In a randomised placebo-controlled study, the drug Chi-Ju-Di-Huang-Wan was used to treat dry eye in 40 subjects, whereas 40 other subjects received a placebo of the same weight, color, and regime without the active drug [15]. In this study, the fluorescein tear break-up time was significantly improved compared to placebo at 4 weeks, whereas Rose Bengal staining, an indicator of corneal epithelial damage, was significantly at 2 weeks compared to placebo. 

 Despite the encouraging results of these modern, properly controlled studies in the treatment of dry eye by TCM, there has been no studies on the awareness of the use of TCM or the extent of practice of TCM in dry eye among TCM practitioners in Singapore. A previous evaluation of knowledge and attitude of TCM in Singapore studied only the parents of children who attended a TCM clinic and pediatricians, without any focus on specific disease conditions [13]. Therefore we aim to conduct a survey of knowledge, attitude, and practice of TCM in treatment of dry eye in institutional TCM practitioners in Singapore.

## 2. Methods

### 2.1. Participants and Inclusion Criteria

Initially we included all current TCM practitioners in Singapore in the study. This involved 2309 participants registered with the Singapore TCM practitioners board (STCMPB), identified from the official on-line database. Out of 2309 participants, 2226 were active and 1929 had a valid, registered address which allows them to be contacted. 

A self-addressed stamped return envelope was enclosed with a questionnaire and mailed out to all eligible participants. The number of returned questionnaires was 407 out of 1929 sent envelopes, accounting for a response rate of 21%. The response rate from institutional practitioners and practitioners with private clinics was much higher 377/564 or 67%, compared to home-based practitioners of 30/1365 or 2%. Therefore the study population was redefined as TCM practitioners working in institutions in Singapore.

The study was approved by the Institutional Review Board (IRB) of the Singapore Eye Research Institute, adhering to the tenets of the Declaration of Helsinki. Informed consent was not obtained from study participants. Since this is a mailed survey, a brief letter that informs participants on their role and the aim of the study is considered sufficient, data were analysed anoymously, and a consent form would be unnecessary. Waiver of consent was approved by the IRB.

### 2.2. Questionnaire Assessment

The detailed questionnaire is provided in the Appendix section. Briefly there were 14 questions, on the knowledge (2 questions), attitude (3 questions), and practice (7 questions) of TCM practice in the treatment of dry eye. There were also two initial questions concerning the workplace and the length of practice of TCM. The questions were translated into Mandarin by a STCMPB-certified TCM practitioner and validated by research staff at Singapore Eye Research Institute. 

### 2.3. Statistical Analysis

The software SPSS for windows was used to evaluate *t*-tests for 2 independent samples with continuous variables. Logistic regression was used to evaluate dichotomous dependent variables, for example, whether participants believe acupuncture can be used for treatment of dry eye. Statistical significance was set at alpha = 0.05. We also recorded individual comments for a qualitative assessment of attitude and practice if these were available.

## 3. Results

### 3.1. Participant Demographics

**Figure 1:**
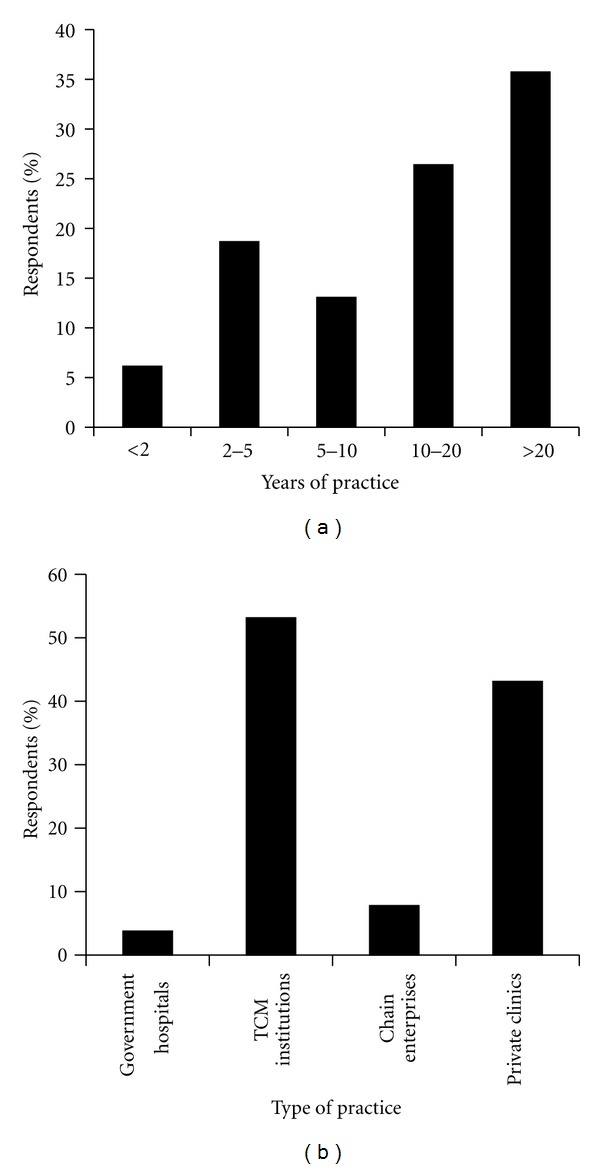
Demographics of surveyed TCM practitioners. Bar chart representing (a) number of years of practice among surveyed TCM practitioners and (b) place of practice of TCM practitioners. “Conventional hospitals” refer to places with mainly conventional medicine and diagnosis, for example, Singapore General Hospital, Tan Tock Seng Hospital; “TCM institutions” are places of TCM clinical practice and education, for example, Thong Chai Medical Institution, Chung Hwa Medical Institution, whereas “chain enterprises” are mostly commercially driven TCM clinics with several branches, for example, Eu Yan Seng, Ma Kuang, Beijing Tong Ren Tang; “private practice” are individual private clinics that usually include 1-2 TCM practitioners; not included are miscellaneous locations not described by above categories: for example, Nanyang Technological University TCM Clinic, clinics at spas and wellness groups, and so forth.

A total of 377 participants responded. There were 41% men and 59% women. The mean age of participants was 56.8 years (SD: 18.0). Slightly more than a third of the respondents had practiced for more than 20 years (Figure 1(a)). About half of these practitioners worked in charity type or nonprofit TCM hospitals and practices, with the other half in private TCM clinics and enterprises such as Eu Yan Seng and Ma Kuang, and spas etc. TCM hospitals include Chung Hwa Medical Institute and Thong Chai Medical Institute. A small minority worked in conventional hospitals such as Singapore General Hospital. Fourteen percent practiced in 2 places and 2% practiced in 3 or 4 places (Figure 1(b)).

### 3.2. Knowledge

**Figure 2:**
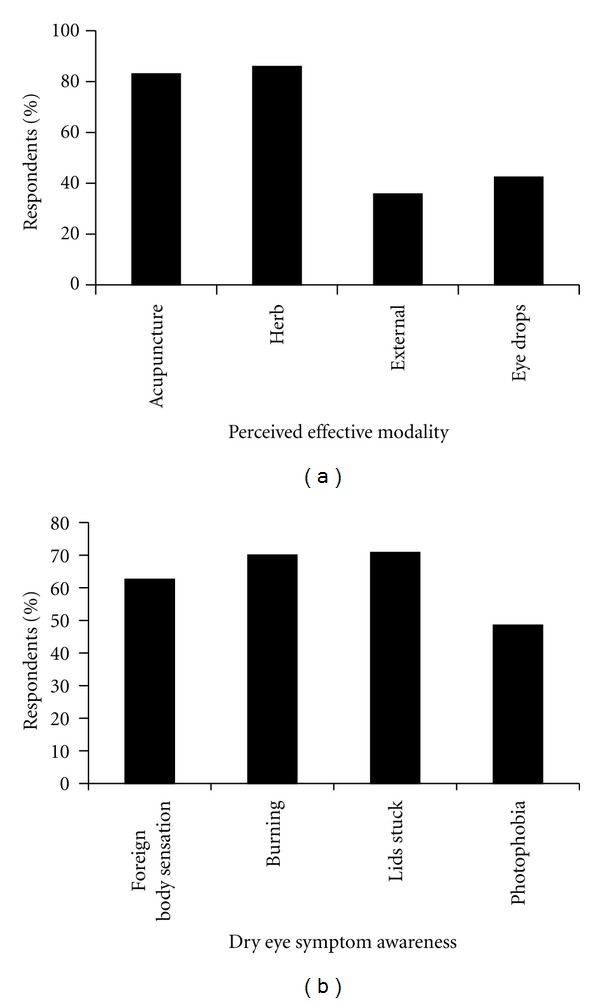
Knowledge of TCM practitioners about dry eye disease. (a) Bar chart representing modality of TCM treatment that practitioners believe to be effective in treating dry eye. “Herbal medication” represents oral herbal decoctions or patent pills; “external treatment” refers to modalities such as hot compress, external herbal wash and eye drops refer to conventional topical eye drops. (b) Bar chart showing the dry eye symptoms that TCM practitioners are aware of.

For the treatment of dry eye, more participants considered the use of herbal and acupuncture modalities to be appropriate as compared to external forms of treatment such as hot compress or external herbal wash, or use of lubricant eye drops. Ninety-five percent of respondents believed that at least one of these two forms of treatment (herbal medication or acupuncture) could be used in dry eye (Figure 2(a)). 

In general the level of knowledge concerning dry eye symptoms was low. Out of 4 symptoms of dry eye evaluated, 30% of the practitioners were aware that all 4 were dry eye symptoms, whereas 19% thought that only 3 of those were dry eye symptoms, and 27% were aware that 2 of those were dry eye symptoms. Nineteen percent of the respondents were aware of only one of those symptoms as part of dry eye disease. Among all respondents, 62–69% knew that foreign body sensation, burning/pain, or eyelids sticking together (at least one of these symptoms) was a symptom of dry eye. Fewer participants (49%) knew that photophobia was a symptom of dry eye (Figure 2(b)). 

### 3.3. Attitude

**Figure 3:**
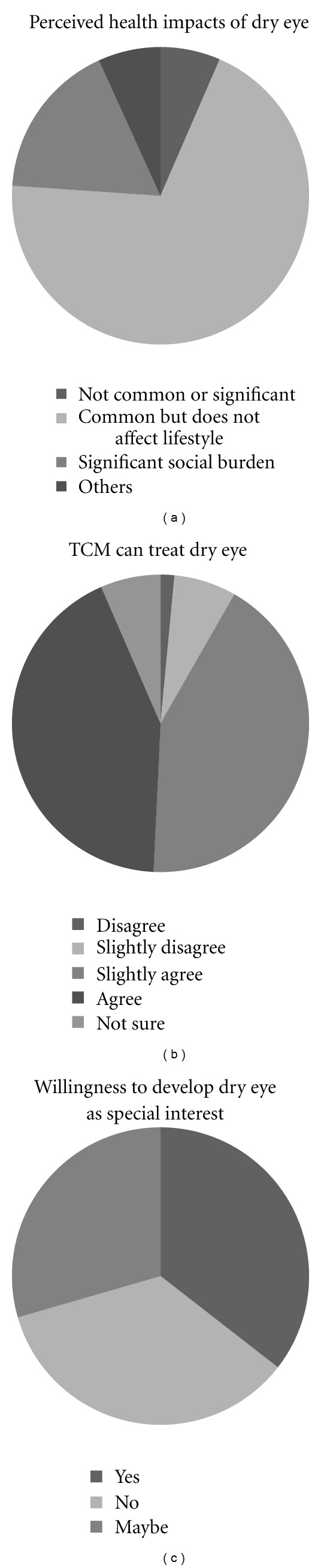
Attitude of TCM practitioners towards dry eye disease. Pie chart illustrating TCM practitioners' perception of (a) dry eye disease, and (b) efficacy of TCM in treatment of dry eye. Disagree: TCM is inappropriate to treat dry eye; slightly disagree: only some patients improve; slightly agree: most (>70%) patients show improvement; strongly agree: dry eyes can be symptomatically cured in most (>70%). (c) Pie chart showing the willingness of TCM practitioners to develop a special interest in treating dry eye.

More than two-thirds of participants thought that dry eye is a common condition and should be treated, but is not severe enough to affect daily activities or not significant enough to be a socioeconomic burden (Figure 3(a)). 

A very high proportion of participants (87%) slightly or strongly believe that TCM can treat dry eye (Figure 3(b)). “Strongly agreeing” was defined as believing 70% or more patients benefitting from treatment. Interestingly, about one-third of the participants indicated that they would consider having “dry eye treatment” as a special area of interest to specialize in, although one-third was against this idea, and one-third were unsure (Figure 3(c)). Many in the latter 2 categories expressed the view that TCM treats patients holistically and specialization in a dry eye is noncompatible or undesirable.

### 3.4. Practice

**Figure 4:**
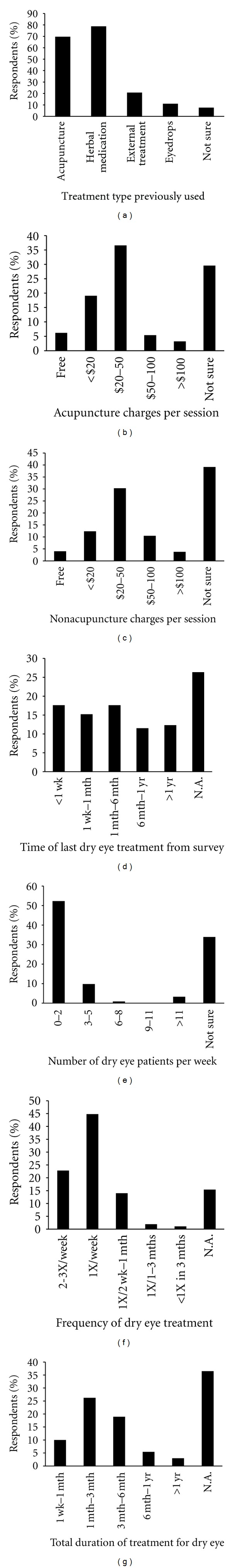
Practice of TCM practitioners in treatment of dry eye disease. Bar chart representing (a) modalities of TCM treatment that practitioners have used to treat dry eye. “Herbal medication” represents oral herbal decoctions or patent pills; “external treatment” refers to modalities such as hot compress and external herbal wash and eye drops refer to conventional topical eye drops, (b) charges for acupuncture treatment per session, (c) charges for nonacupuncture TCM treatment per session, (d) period of time from the last TCM treatment of a dry eye patient, (e) average number of dry eye patients treated per week by TCM practitioners, (f) frequency of TCM treatment and follow-up visits for dry eye patients, and (g) entire span of TCM treatment course for dry eye patients. Wk: week, Mth: month. All charges are in Singapore currency.

Eighty-five percent of the respondents indicated experience with treating dry eye. Amongst those with dry eye treatment experience, a high proportion of respondents have performed acupuncture or herbal medicine treatment (71% and 80% resp.). Twenty-one percent have used external treatments and 11% eye drops (Figure 4(a)).

The cost or charge of acupuncture is usually S$20–50 per session (Figure 4(b)) and the cost of nonacupuncture treatment, including predominantly herbal medication, is also usually S$20–50 per consultation (Figure 4(c)). The majority of respondents stated that average frequency of treatment that they implement for dry eye is 1–3 times a week and the total duration of treatment is 1–6 months. One US$ is equivalent to about S$1.26.

Most respondents have fairly recent experience with treatment of dry eye. The time of the most recent consult was widely spread, with 15–20% of participants managing the dry eye patient either less than one week ago, between one week and one month ago, or between one and six months ago. A further 11% saw the last patient with dry eye between six months and one year ago, and another 12%, more than one year ago (Figure 4(d)).

On the whole, dry eye patients do not form the bulk of TCM practice among these respondents. Regarding the dry eye burden encountered by each TCM physician, 52% of participants treated 2 or less patients per week on average (Figure 4(e)), although 3% treated more than 11 patients in a week. In general the most common frequency of treatment was once a week (Figure 4(f)) and the total span of the treatment course was most commonly between one to six months (Figure 4(g)).

We next explore if the gender, age, years of practice, or place/type of practice were associated with the knowledge, attitude, and practice of TCM practitioners in dry eye. The most statistically significant findings are as follows. 

Practitioners who have worked less number of years compared to those who worked for more years believe that acupuncture treatment is appropriate for dry eye (*P* < 0.001) and actually perform acupuncture for dry eye (*P* = 0.009). Practitioners who have worked less number of years compared to those who worked for longer durations are more likely to think that external treatment can be used for dry eye (*P* = 0.001) and actually used external treatment (*P* = 0.001) for dry eye more often.

### 3.5. Qualitative Analysis

Some participants were very responsive and wrote comments or attached appendices with completed questionnaires. This section summarizes these comments. 

Unlike conventionally trained medical doctors, TCM practitioners in Singapore rarely specialize in diseases and “TCM ocular specialists” are few and far between. We received feedback (3 responses) that most patients in TCM clinics present with common colds, cough, abdominal pain, nausea, and so forth, and dry eye patients are rare. Often, dry eye is presented as an accompanying symptom and patients do not usually seek medical care for primarily dry eye disease (6 responses), consistent with a report in the USA [3]. As such, the awareness for its severity is low. Also, many practitioners believe that TCM is a form of holistic medicine and that “dry eye disease” should not be singled out as a “specialty” (27 responses). As such, many were unwilling to develop a special interest in dry eye. 

Generally, the majority of respondents are positive about the efficacy of TCM in treating dry eye. A few wrote about their personal experience of having their own dry eyes treated with TCM despite having had a chronic history (2 responses), and some wrote about their successful cases (3 responses). Some respondents commented that dry eye is difficult to cure and TCM is not effective in alleviating symptoms of dryness (2 responses).

## 4. Discussion

Three quarters of respondents have previously treated dry eye. About three quarters of respondents believe that dry eye was not severe enough to be a public health burden but most think that TCM is effective in the treatment of dry eye. Acupuncture and herbal medicine are the most commonly used modalities in dry eye treatment, and treatment with eye drops is relatively uncommon. One-third of respondents would consider developing an interest in the treatment of dry eye.

### 4.1. Comparison with Other Studies

There has not been any published study on the knowledge, attitude, and practice of TCM in dry eye treatment. Only 2 papers on the attitude of health professionals towards dry eye treatment have been published previously [25, 26]. Both studies surveyed predominantly UK and Australian ophthalmologists and optometrists on their attitudes toward dry eye and diagnostics and therapies. The participants in both studies showed a lack of interest and underestimation of the severity of dry eye, as well as dissatisfaction with available diagnostics and therapies. Jeon et al. reported that “eye practitioners are hopeful for a breakthrough in diagnosis and treatment options” [26].

The results and implications of previous studies may provide some insight to conventional diagnostics and treatments for dry eye disease; however they cannot be extrapolated to complementary and alternative medicine practices such as TCM. The fundamental principles and treatments are vastly different in conventional medicine and TCM and as such, knowledge, attitude, and practice of TCM practitioners will most likely differ from conventional ophthalmologists.

### 4.2. Limitations to Our Study

We did not include home TCM practitioners in this study, only targeting 576 out of 1929 or 30% of the registered practitioners. The respondents working in institutions may or may not be representative of the general TCM community in Singapore. It is not possible to determine the attitudes of the home practitioners and in fact, with no official employer, there is no way to ascertain if they still practice. The response rate for the study (67%) on institutional practitioners is expected and acceptable [25, 26]. Nevertheless, there may be selection bias in that respondents with a higher interest in dry eye would be more likely to respond to the survey. 

### 4.3. Clinical Application and Future Studies

Dry eye disease is a prevalent condition in adult populations and accounts for significant healthcare resources in developed countries [3]. Current mainstream treatment of dry eye involves predominantly the usage of topical eye drops which usually provides only temporary symptomatic relief. Compliance is a major issue as patients find continued treatment to be ineffective, costly, or troublesome. As such, alternative treatments for dry eye disease should be explored. Our study found that a considerable number of TCM practitioners believe TCM to be effective in managing dry eye and have had experience of treating dry eye. 

There are published clinical trials on the efficacy of TCM treatment (acupuncture [16–20, 27] and herbal medication [15, 27]). Shin et al. [20] developed a sham acupuncture system for a randomized controlled trial. The lack of a proper sham acupuncture control is a major limitation in acupuncture studies in the past [28]. Chang et al. [15] used placebo tablets as a control for Chi-Ju-Di-Huang-Wan, a Chinese patent drug commonly used in dry eye treatment. Shin et al. showed that tear break-up time can be improved by acupuncture [20, 29] whereas Chang showed that tear break-up time and Rose Bengal staining were improved by herbs [15]. Further studies on the efficacy and safety of specific TCM modalities in dry eye have been planned [10, 25]. The ubiquitous use of alternative medicine in Singapore [12] suggests that health planners, policy makers, and the medical profession need to consider how it can be integrated into healthcare. More efficacy and safety studies on specific types of TCM should be conducted.

The average cost of TCM treatment, both acupuncture and herbal medication, is about $20–50 per session per episode, including consultation fees. The majority of respondents stated that average frequency of treatment that they implement for dry eye is 1–3 times a week and the total duration of treatment is 1–6 months. The cost of TCM appears reasonable compared to per physician consultation of $60–100 and additional topical lubricants, gels, and immunosuppressives such as cyclosporine emulsions, with the latter costing about $70 for 3 weeks [30], driven by hospital-based ophthalmic care [22, 24]. In the dry eye clinic in one of the authors at Singapore National Eye Center, patients tend to seek consultation 1–4 times a year for up to a few years (unpublished data). Such visits are known to incur considerable costs [22, 24, 31]. 

A high proportion of respondents acknowledge that dry eye is a common problem though not severe enough to affect daily activities and unaware of all the symptoms that dry eye presents. The lack of knowledge may be related to a lack of emphasis on these aspects in TCM education. We should promote greater awareness of the importance of dry eye amongst TCM practitioners.

Further studies should be conducted to delineate the exact effectiveness, cost benefit, and role of TCM treatment in the entire health care process. It would be useful to survey patients who have received TCM treatment in addition to topical eye drops regarding its perceived cost and efficacy. 

## 5. Conclusion

A considerable number of institutional TCM practitioners have previously treated dry eye with acupuncture or herbal treatment and are convinced of the effectiveness of TCM treatment in managing the condition. Most respondents charge lower rates for their treatment which lasts over a period of 1–6 months. A significant proportion of respondents is keen to pursue specialized treatment in dry eye. While the knowledge of dry eye symptoms is generally not high, this could be due to differences in dry eye definition across the two different medical systems.

## Appendix

Gender: Male/female
Date of birth: ___________________ (dd, mm, yyyy)
(1) For how long have you practiced Chinese Medicine?
  1. <2 years
  2. 2–5 years
  3. 5–10 years
  4. 10–20 years
  5. 20 years
(2) Where do you mainly practice TCM?
  1. Government, restructured hospital, or private hospital (e.g., SGH, TTSH, Raffles Medical Group)
  2. TCM hospital (e.g., Thong Chai Medical Institution, Chung Hwa Medical Institution)
  3. TCM group practice (e.g., Eu Yan Sang, Ma Guang)
  4. Private clinic (1-2 locations)
  5. Home practice
  6. Others, please specify: ____________________________________________
(3) Dry eye can be treated with the following:
  1. Acupuncture
  2. Herbal decoctions taken orally
  3. Herbal eye wash or hot compress
  4. Western topical eye drops
  5. Not sure
(4) Dry eye may present the following symptoms:
  1. Grittiness of eye
  2. Burning sensation or pain in eye
  3. Tiredness in eyes or eyelids sticking together
  4. Phobia of light
  5. Not sure
(5) To me, dry eye is:
  1. Not a common or significant condition
  2. Common but does not affect daily activities
  3. A significant health problem; needs a lot of health resources
  4. Others, please specify: _________________________________________
  5. Not sure
(6) TCM can be used to improve dry eye.
  1. Disagree: other forms of treatment are required to cure dry eye
  2. Slightly disagree: only some people improve
  3. Slightly agree: most (>70%) of them only show improvement
  4. Strongly agree: dry eyes can be symptomatically cured in most (>70%)
  5. Not sure
(7) Are you willing to see patients with primarily dry eye in your practice in future?
  1. Yes
  2. No
  3. Maybe, please specify reason: _____________________________________
(8) What treatments for dry eye have you performed?
  1. Acupuncture
  2. Herbal decoctions taken orally
  3. Herbal eye wash or compress
  4. Western topical eye drops
  5. Not applicable
(9) What is the estimated cost of the treatment per session if acupuncture is performed?
  1. Free
  2. <$20
  3. $20–$50
  4. $50–$100
  5. >$100
  6. Not applicable
(10) What is the estimated cost of treatment per session if acupuncture is not performed?
  1. Free
  2. <$20
  3. $20–$50
  4. $50–$100
  5. >$100
  6. Not applicable
(11) When was the last time you treated a patient with dry eye?
  1. Between today and one week ago
  2. Between 1 week and 1 month ago
  3. Between 1 month and 6 months ago
  4. Between 6 months and 1 year ago
  5. More than 1 year ago
  6. Not applicable
(12) On the average, how many dry eye patients do you treat?
  1. <2 patients every week
  2. 3–5 patients every week
  3. 6–8 patients every week
  4. 9–11 patients every week
  5. >11 patients every week
  6. Not sure
(13) What is the average frequency of followup for the treatment course of dry eye?
  1. 2-3 times a week
  2. Once a week
  3. Once a fortnight to once in a month
  4. Once a month to once in 3 months
  5. Less than once in 3 months
  6. Others: ____________________
(14) What is the entire span of each treatment course?
  1. Between 1 week to 1 month
  2. Between 1 month to 3 months
  3. Between 3 months to 6 months
  4. Between 6 months to 1 year
  5. More than 1 year
  6. Others: _______________________
